# RNF43 is associated with genomic features and clinical outcome in BRAF mutant colorectal cancer

**DOI:** 10.3389/fonc.2023.1119587

**Published:** 2023-06-20

**Authors:** Peipei Shang, Jiongjiong Lu, Feihong Song, Yijun Zhao, Weipeng Hong, Yuange He, Weidong Shen, Li Geng

**Affiliations:** ^1^ Oncology Department, Eastern Hepatobiliary Surgical Hospital, Shanghai, China; ^2^ Department of Special Treatment, Eastern Hepatobiliary Surgical Hospital, Shanghai, China; ^3^ Department of Medical Center, Geneplus-Beijing Ltd., Beijing, China

**Keywords:** colorectal cancer, RNF43, BRAF, genomic features, clinical outcome

## Abstract

**Background:**

Colorectal cancer (CRC) patients with BRAF mutation have very poor prognosis. It is urgent to search for prognostic factors of BRAF mutant CRC. RNF43 is a ENF ubiquitin ligase of Wnt signaling. Mutation of RNF43 has been observed frequently in various types of human cancers. However, few studies have evaluated the role of RNF43 in CRC. The present study aimed to explore the impact of RNF43 mutations on molecular characteristics and prognosis in BRAF mutant CRC.

**Methods:**

Samples of 261 CRC patients with BRAF mutation were retrospectively analyzed. Tumor tissue and matched peripheral blood samples were collected and subjected to targeted sequencing with a panel of 1021 cancer-related genes. The association of molecular characteristics and survival in patients were then analyzed. 358 CRC patients with BRAF mutation from the cBioPortal dataset were used for further confirmation.

**Results:**

This study was inspired by a CRC patient with BRAF V600E and RNF43 co-mutation, who achieved a best remission of 70% and a progression free survival (PFS) of 13 months. Genomic analysis indicated that RNF43 mutation affected the genomic characteristics of patients with BRAF mutation, including microsatellite instability (MSI), tumor mutation burden (TMB) and the proportion of common gene mutations. Survival analysis showed that RNF43 mutation was a predictive biomarker for better PFS and OS in BRAF mutant CRC.

**Conclusion:**

Collectively, we identified that RNF43 mutations were correlated with favorable genomic features, resulting in a better clinical outcome for BRAF mutant CRC patients.

## 1 Introduction

Colorectal cancer (CRC) is one of the most common cancers with the third cancer-related lethal rate worldwide (1). Despite the great progress in cancer research of the last decade, the prognosis of metastatic CRC (mCRC) patients remains poor with a 5-year overall survival (OS) rate of approximately 14%. Thus, more in-depth research at molecular level were conducted to have a better understanding of this disease. Like many malignant cancers, CRC is a heterogeneous disease with several subtypes characterized by genetic alterations. BRAF gene mutation occurred in approximately 12% of CRC patients, and the majority subtype is the missense of V600E mutation. The BRAF mutation associated with various clinical features was well studied, it was more common in women older than 70, more common for tumors located in the right colon, more common in mucinous subtype, about 60% of the tumors are poorly differentiated (2–5).

Several clinical studies have revealed mCRC patients with BRAF mutation had a poor survival relative to that wild type patients, the median overall survival (mOS) was 2-3 years versus 1 year (5, 6). For patients diagnosed at early stage, curative surgery was preferred, however, the treatment for mCRC patients was based largely on systemic chemotherapy and the optimal first-line therapy for BRAF mutant mCRC patients remains unclear. The TRIBE study evaluated first-line chemotherapy in mCRC, the patients harboring BRAF V600E mutations treated with FOLFOXRI plus bevacizumab in TRIBE trial achieved longer survival than treated with FOLFIRI plus bevacizumab (mOS: 19months vs. 10.7 months), and the mOS in the RAS and RAF WT subgroup was 37.1 months versus 13.4 months in BRAF WT subgroup, highlight again the poor outcomes associated with BRAF mutation (7). In clinical, the TNM system was widely used for risk assessment and therapy decision making. However, due to the high level of molecular heterogeneity, the patients’ clinical benefit and survival may be various even in the same clinicopathological features. Hence, novel prognostic factors to accurately revealing CRC patients’ survival was needed.

In this context, we reported a case of mCRC harbored BRAF V600E co- mutant with RNF43 that achieved a best remission of 70% compared to baseline and a progression free survival (PFS) of more than 13 months after receiving first-line chemotherapy therapy. With this case, we propose that RNF43 mutation may affect the prognosis of BRAF mutant CRC, and through the exploration of data from the expanded samples and cBioPortal, we draw a conclusion that RNF43 mutation may have a favorable prognostic effect in BRAF mutant CRC population.

## 2 Materials and methods

### 2.1 Patients and clinical tissues

The present study retrospectively enrolled 261 BRAF mutant CRC patients who underwent an NGS assay in Geneplus-Beijing Co. Ltd. (Beijing, China) between March 2016 and April 2022 (**Supplementary Table 4**). The study was approved by the Ethics Committee of Eastern Hepatobiliary Surgical Hospital (No. EHBHKY2018-02-023). All patients provided written consent for genetic analysis. Fresh or formalin-fixed paraffin-embedded (FFPE) tissues and matched peripheral blood (PBL) samples were collected and sent to geneplus for genetic testing.

### 2.2 Genetic profiling

Genetic profiling was performed using targeted next generation sequencing (NGS) as previously described (8, 9). In brief, peripheral blood lymphocytes (PBL) was separated with centrifugation. Genomic DNA from PBLs and tumor tissues were separately obtained using the DNeasy Blood & Tissue Kit (Qiagen, Hilden, Germany) and sheared. Sequencing libraries were generated with the KAPA DNA Library Preparation Kit (Kapa Biosystems, Wilmington, MA, USA). Barcoded libraries were hybridized to a custom-designed panel containing 1021 cancer-related genes (**Supplementary Table 2**). DNA sequencing was performed on the Geneplus-2000 Sequencing platform or HiSeq 3000 instrument according to the manufacturer s protocol. Targeted capture sequencing required a minimal mean effective depth of 500 in tissue DNA. Single nucleotide variants (SNVs) were called using MuTect (version1.1.4), and small insertions and deletions (indels) were called by GATK. Copy number variations (CNVs) were detected using Contra (2.0.8), and structural variations (SVs) were detected using BreakDancer. Germline variants in PBL DNA samples were identified according to the Single Nucleotide Polymorphism database (dbSNP). The somatic mutations in tumor tissues were further confirmed with the following criteria: (a) variants occur at <1% of the population frequency in the 1000 Genomes Project and the Exome Aggregation Consortium; (b) absent in paired germline DNA from PBLs; and (c) present in greater than or equal to five high quality reads (Phred score 30, mapping quality 30), and without paired-end reads bias. MSI status was defined as MSI-H or microsatellite stable (MSS) using MSI sensor (v0.2). Tumors with an MSI score ≥10 were classified as MSI-H. Patients harboring ≥20 mutations/megabases (Mb) were classified as TMB-H, while those with <20 mutations/Mb were TMB-low (TMB-L).

### 2.3 Statistical analysis

CRC dataset was downloaded from the cBioPortal website (http://www.cbioportal.org/, accessed on 9 March 2021). Categorical variables were assessed using the chi-square test or Fisher’s exact test. Differences between the two groups were examined using a two-tailed unpaired Mann-Whitney test. Statistical analyses were performed using the Prism analysis and graphics software (GraphPad) version 8.0.2. The Maftool package, an R Bioconductor package, was used to analyses genetic aberrations in different pathways (10). A two-sided P value of < 0.05 was considered statistically significant.

## 3 Results

### 3.1 Case

**Figure 1:**
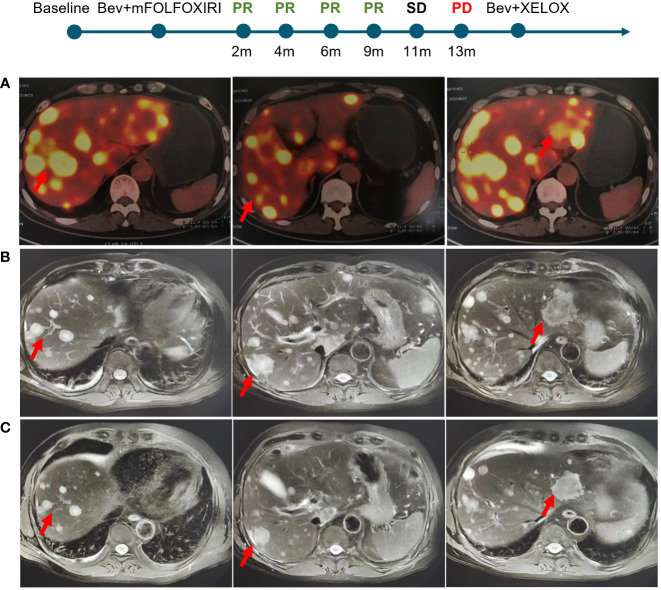
Imaging changes of liver metastatic lesions of the patient. **(A)** Baseline. **(B)** 4 cycles after mFOLFOXIRI-bevacizumab treatment. **(C)** 8 cycles after treatment.

A 60-years-old man presented with abdominal discomfort in September 2020, accompanied by vomiting and constipation. B-ultrasound was performed and hepatic space-occupying lesion was identified. Positron emission tomography computed tomography (PET-CT) showed irregular thickening of the intestinal wall in the middle and lower segment of the ascending colon accompanied by luminal narrowing and increased 18F-fluoro-2-deoxy-d-glucose (18FDG) uptake. It was considered that colon cancer involved serosal surfaces, multiple lymph node metastases in the mesentery, retroperitoneum and hepatic hilum, pelvic floor membrane implantation metastases, and multiple metastases in the liver (the largest was about 75mm in diameter, **Figure 1A**). On September 30, 2020, colonoscopy and liver biopsy were performed and the pathological diagnosis was moderately differentiated adenocarcinoma. The patient was treated with mFOLFOXIRI-bevacizumab on October 2, 2020 after genetic testing revealed BRAF V600E mutation in the tumor tissue. After 4 cycles of chemotherapy, computed tomography (CT) revealed the tumor was reduced by about 50% (**Figure 1B**) and the response was evaluated as PR (RECIST v1.1). CT was conducted again on January 23, 2021, the tumor was reduced by 28% compared with last time and 70% reduction from baseline (**Figure 1C**), The response was evaluated as PR. Considering the patient’s physical condition, the chemotherapy regimen was changed to Capecitabine and Bevacizumab on March 27, 2021. The response evaluation in March, June and October was PR, SD without enlargement and PD, respectively. On October 23, 2021, the regime was changed to XELOX and Bevacizumab, and the patient was still alive now. Overall, this patient with mCRC harboring BRAF V600E mutation responded significantly after receiving triplet mFOLFOXIRI plus bevacizumab chemotherapy regimen, with an optimal response of 70% tumor reduction and achieved a PFS of 13 months.

### 3.2 Effects of RNF43 mutation on BRAF mutant CRC

**Figure 2:**
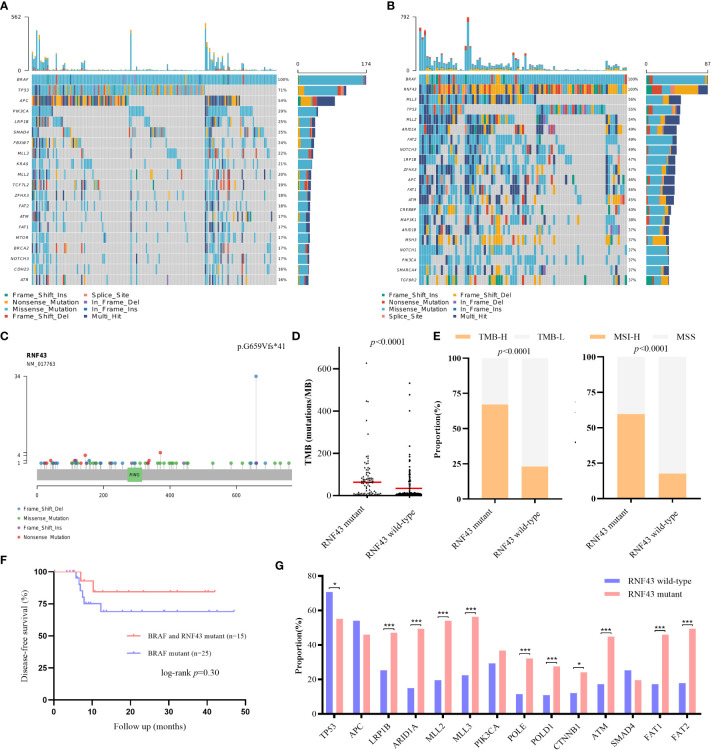
Differences of genomic features and survival between RNF43 wild-type and mutant group in BRAF mutant retrospective cohorts. **(A, B)** Landscape of genetic mutations in 174 RNF43 wild-type and 87 RNF43 mutant samples. **(C)** Mutation spectrum of RNF43. **(D)** DFS between patients with or without RNF43 mutation. **(E)** TMB value and status in RNF43 wild-type and mutant group. **(F)** MSI status in RNF43 wild-type and mutant group **(G)** Frequency of common altered genes in RNF43 wild-type and mutant group. * means p<0.05, ** means p<0.01, *** means p<0.001.

RNF43 has been identified as a possible oncogenic gene in previous studies. Based on the patient’s genetic testing results, we speculated that RNF43 mutation might affect the prognosis of BRAF mutant CRC patients. To verify the hypothesis, we retrospectively collected a cohort of 261 CRC samples harboring BRAF mutation and divided them into two groups: 174 patients in the RNF43 wild-type group and 87 patients in the RNF43 mutant group (**Supplementary Table 1**). The baseline clinical characteristics were summarized in **Table 1**. All samples were subjected to NGS using a panel of 1021 cancer related genes as previously reported. The lollipop chart showed that most RNF43 mutations in BRAF mutant CRC samples were frame-shift subtypes, among which the p.G659Vfs *41 mutation accounted for the highest frequency of 39% (**Figure 2C**). RNF43 mutant group had more mutations compared with the wild-type group. The most common mutant genes between the two groups were significantly different. The top five genes with the highest mutation rates in RNF43 mutant group were MLL3, TP53, MLL2, ARID1A, FAT2, the wild-type group were TP53, APC, PIK3CA, LRP1B, SMAD4 (**Figures 2A, B**). Then we followed up 25 RNF43 wild-type and 15 RNF43 mutant patients, the baseline clinical characteristics were summarized in **Table 2**. There was no statistical difference in disease-free survival (DFS), possibly because of too few outcome events so far (**Figure 2F**). Further analysis of the genomic features of the two groups showed that the TMB value (median TMB: 10.56 mutations/Mb versus 9.6 mutations/Mb, p<0.0001), TMB-H ratio (67.1% versus 23%, p<0.0001) and MSI-H ratio (59.7% versus 17.7%, p<0.0001) of RNF43 mutant group were significantly higher than those of wild-type group (**Figures 2D, E**). Finally, we compared the mutation rates of key driver genes and proven favorable genes in CRC, and found that the mutation rate of most genes in RNF43 mutant group was significantly higher than that in wild-type group (**Figure 2G**). In conclusion, the genomic features of the RNF43 mutant group were significantly different from those of the RNF43 wild-type group, which could be reflected in TMB, MSI and gene mutation rates.

**Table 1:** Clinical characteristics of 261 BRAF mutant CRC patients.

	RNF43 wild-type N=174	RNF43 mutant n=87
Age	59(24-88)	58(32-85)
Sex	17(NA)	10(NA)
Male	89	47
Female	68	30
Location	37(NA)	41(NA)
L	79	15
R	34	20
Rectal	24	11
TMN	32(NA)	29(NA)
I-III	72	32
IV	70	26

**Table 2:** Clinical characteristics of 40 follow-up patients.

	RNF43 wild-typeN=25	RNF43 mutantn=15
Age	56(33-83)	65(36-76)
Sex		
Male/Female	16/9	10/5
Location		
L	13	3
R	6	10
Rectal	6	2
TMN		
I	3	2
II	13	7
III	7	5
IV	2	1
Adjuvant therapy		
Yes	13	8
No	7	6
NA	5	1

### 3.3 Analysis of genomics in public BRAF mutant cohorts

**Figure 3:**
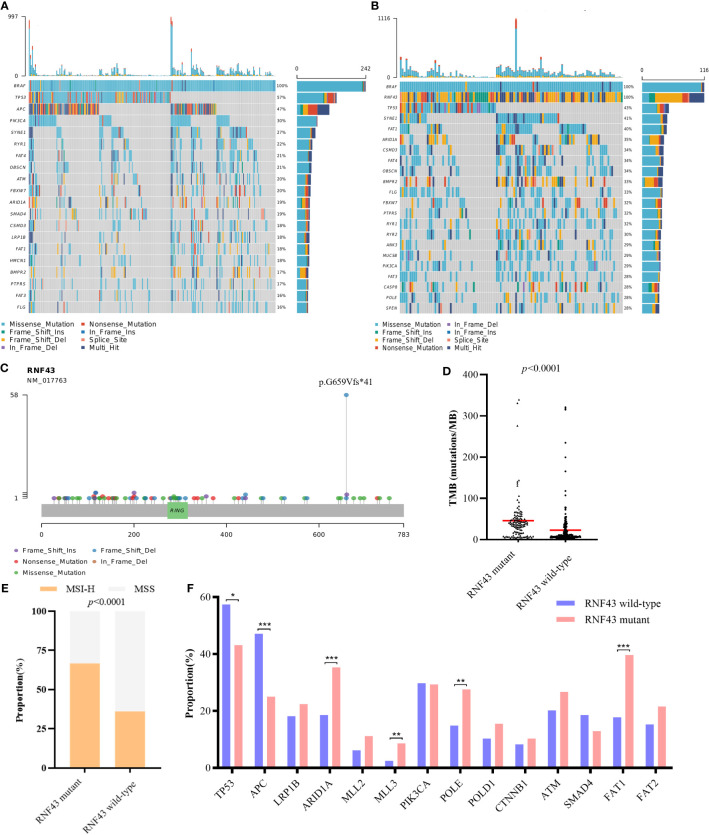
Differences of genomic features between RNF43 wild-type and mutant group in BRAF mutant public cohorts. **(A, B)** Landscape of genetic mutations in 242 RNF43 wild-type and 116 RNF43 mutant samples. **(C)** Mutation spectrum of RNF43. **(D)** TMB value in RNF43 wild-type and mutant group. **(E)** MSI status in RNF43 wild-type and mutant group **(F)** Frequency of common altered genes in RNF43 wild-type and mutant group. * means p<0.05, ** means p<0.01, *** means p<0.001.

To further verify the prognostic influence of RNF43 mutation in BRAF mutant CRC patients, we collected a total of 358 BRAF mutant CRC samples from 6 cohorts in cBioPortal (coad_cptac_2019, coadread_tcga_pan_can_atlas_2018, coadread_tcga_pub, coadread_tcga, crc_apc_impact_2020 and crc_msk_2017), including 116 samples with RNF43 mutation and 242 samples with RNF43 wild-type (**Supplementary Tables 3, 4**). First, similar to our cohort, RNF43 mutation mainly appeared in the form of frame-shift mutation, and p.G659Vfs*41 mutation accounted for the highest proportion of 50% (**Figure 3C**). Second, mutations in the RNF43 mutation group were more than wild-type group, and the most common mutant genes in the two group were significantly differenced. The five genes with the highest mutation rates in RNF43 mutant group were TP53, SYNE1, FAT1, ARID1A, CSMD3, the wild-type group were TP53, APC, PIK3CA, SYNE1, RYR1 (**Figures 3A, B**). We then analysed the main genomic features TMB and MSI, and found that the TMB value (median TMB: 68.16 mutations/Mb versus 7.68 mutations/Mb, p<0.0001) and the proportion of MSI-H (42.5% versus 17.3%, p<0.0001) in the RNF43 mutant group were significantly higher than those in the wild-type group (**Figures 3D, E**). Finally, there were significant differences in the mutation rates in TP53, APC, ARID1A, MLL3, POLE and FAT1 between the two groups (**Figure 3F**). In summary, as we concluded above, the genomic characteristics of the RNF43 mutant group are significantly different from those of the wild-type group.

### 3.4 Clinical outcomes of public BRAF mutant cohorts

**Figure 4:**
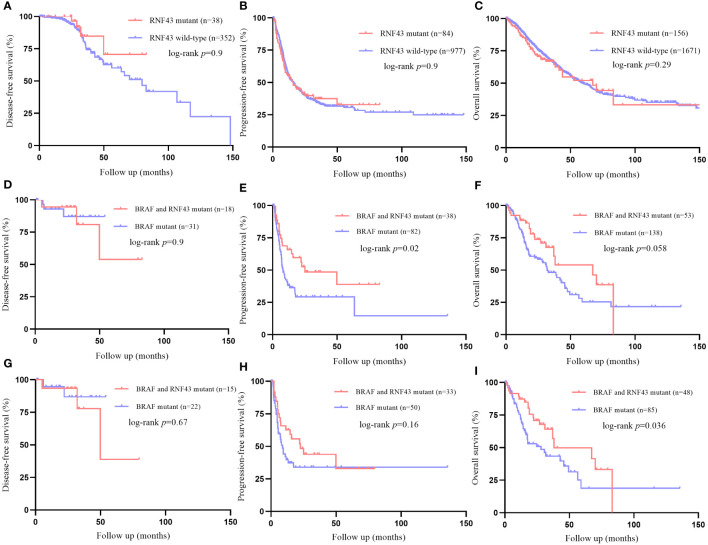
Differences of survival between RNF43 wild-type and mutant group in public BRAF mutant cohorts. **(A–C)** DFS, PFS, OS between patients with or without RNF43 mutation in unselected CRC patients. **(D–F)** DFS, PFS, OS between patients with or without RNF43 mutation in BRAF mutant CRC patients. **(H–J)** DFS, PFS, OS between patients with or without RNF43 mutation in BRAF V600E mutant CRC patients.

In order to further confirm the impact of RNF43 mutation on the prognosis of CRC, we analyzed DFS, PFS and OS in all CRC population of above cBioPortal cohorts, and repeated sample were filtered (**Supplementary Tables 5, 6**). There were no statistically significant differences in DFS, PFS or OS between RNF43 mutations and wild-type group in CRC population (**Figures 4A–C**). This indicates that RNF43 mutation has no prognostic effect on CRC patients without selection. We then selected BRAF mutant population from above cohorts and found that the RNF43 mutation group presented longer PFS and OS. In DFS, there was no statistical difference, possibly due to the small sample sizes (**Figure 4D**). The baseline clinical characteristics were summarized in **Table 3**. mPFS was 25.0 months in the RNF43 mutant group compared with 8.0 months in the wild-type group (HR 0·56, 95% CI 0·34–0·91; p=0·019; **Figure 4E**). mOS was 67.3 months in the RNF43 mutant group compared with 31.9 months in the wild-type group (HR 0·62, 95% CI 0·40–0·97; p=0·058; **Figure 4F**). This was longer than previous studies because not only mCRC patients were included. In view of the predominance of V600E in BRAF mutation and the difference in prognosis, we screened the BRAF V600E population separately and performed corresponding prognostic analysis. We found that the result was similar that RNF43 mutation group presented longer PFS, OS and no statistical difference for DFS (**Figures 4G–I**). However, there was a difference in P-value that OS (HR 0·58, 95% CI 0·35–0.94; p=0·036; **Figure 4J**) was more significant and PFS became insignificant ((HR 0·67, 95% CI 0·38–1.17; p=0·16; **Figure 4I**). These results suggest that RNF43 mutation has a specific effect on the prognosis of BRAF mutant CRC patients.

**Table 3:** Clinical characteristics of 191 patients included in survival analysis.

	RNF43 wild-typeN=138	RNF43 mutantn=53
Age	60(28-89)	75(35-90)
Sex		
Male/Female	71/67	20/33
Location		
L	40	6
R	41	20
Rectal	4	0
NA	53	27
TMN		
I	2	2
II	3	7
III	15	12
IV	36	8
NA	82	24

### 3.5 Effects of ZNRF3 mutation on BRAF mutant CRC

**Figure 5:**
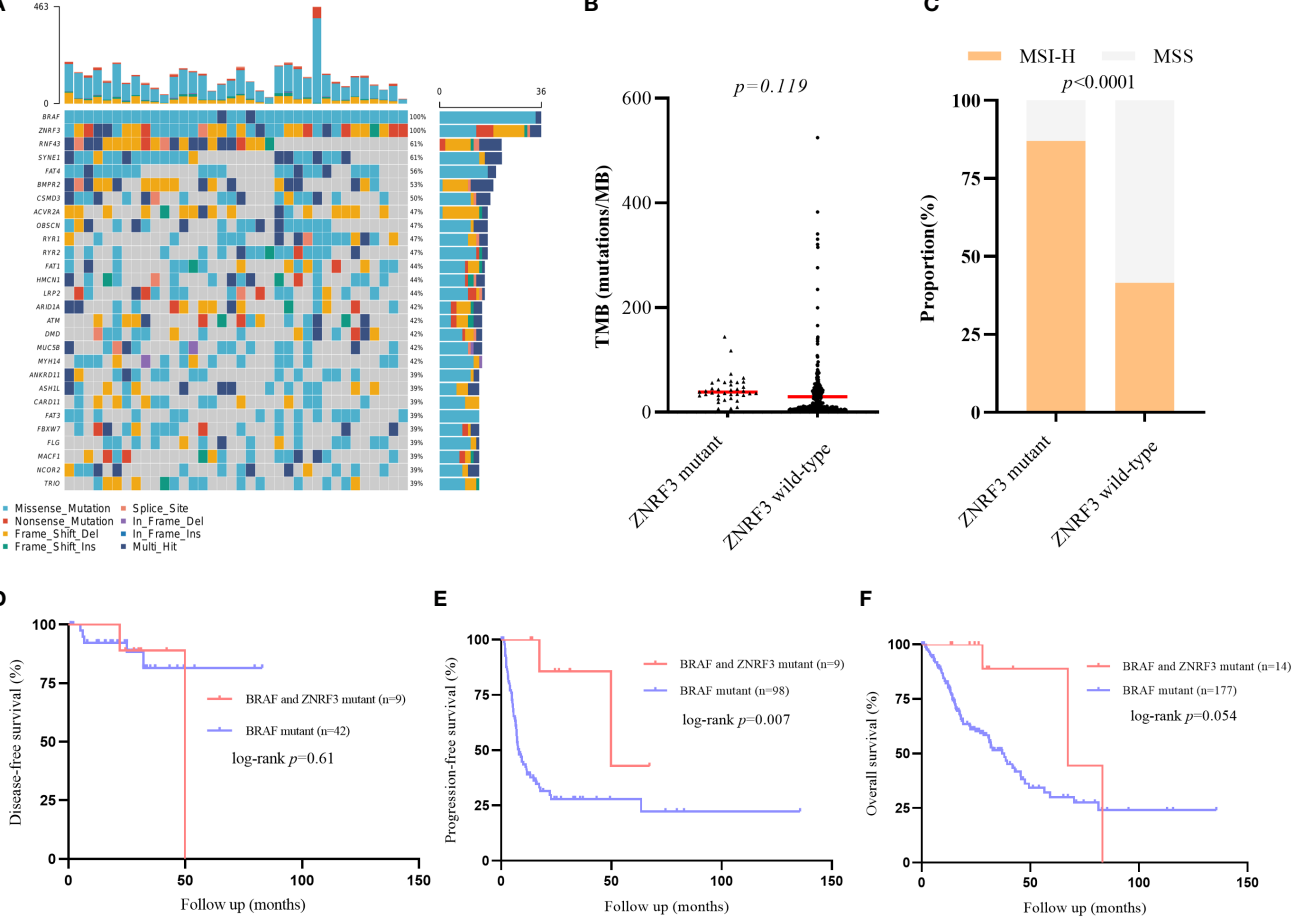
Differences of genomic features and survival between ZNRF3 wild-type and mutant group in public BRAF mutant cohorts. **(A)** Landscape of genetic mutations in 39 ZNRF3 mutant samples. **(B)** TMB value in ZNRF3 wild-type and mutant group. **(C)** MSI status in RNF43 wild-type and mutant group. **(D–F)** DFS, PFS, OS between patients with or without ZNRF3 mutation in BRAF mutant CRC patients.

Previous studies have found that ZNRF3 and RNF43 have similar functions in WNT signaling pathway and carcinogenic mechanisms, we also conducted corresponding analysis on the impact of ZNRF3 mutation in BRAF mutant CRC population from cBioPortal cohorts (**Supplementary Table 7**). Notably, 61% of the ZNRF3 mutant samples carried the RNF43 mutation, further confirming the correlation between the two genes (**Figure 5A**). The TMB values between the two groups were compared and showed no significant difference, which may need more samples to confirm (**Figure 5B**). However, similar to the results of RNF43, there were significant differences in MSI-H ratio between the ZNRF3 mutant group and the wild-type group (**Figure 5C**). The DFS of the two groups was no statistical difference (**Figure 5D**). PFS and OS of the ZNRF3 mutant group were significantly longer than those of the wild-type group. mPFS was 49.8 months in the ZNRF3 mutant group compared with 8.0 months in the wild-type group (HR 0·18, 95% CI 0·09–0·36; p=0·007; **Figure 5E**, **Supplementary Tables 8, 9**). mOS was 67.3 months in the ZNRF3 mutant group compared with 37.0 months in the wild-type group (HR 0·34, 95% CI 0·17–0·70; p=0·054; **Figure 5F**). These results also demonstrated the positive effect of RNF43 mutation on BRAF mutant CRC population.

## 4 Discussion

mCRC patients with BRAF mutation were proved having a poor survival time. Many prognostic biomarkers have been studied in CRC, but rare in BRAF mutant CRC patients. Previous researches showed that CA19-9 and non-V600E mutation may be prognostic marker for BRAF mutant CRC patients (4, 11). Current guidelines recommend first-line intense chemotherapy-bevacizumab for BRAF mutant mCRC patients, but the response is poor and it progressed rapidly. New targeted therapies are also taken into considered, including the RAF inhibitors alone or in combination with MEK inhibitors or anti-EGFR mAb (6). Therefore, as with the development of new treatment options, it is also important to explore effective biomarkers related to the prognosis of the BRAF mutant CRC population. In this study, we report a BRAF V600E mutant mCRC patient had a PFS of 13 months after first-line standard therapy, with an optimal response of 70% reduced PR, and was alive up to now.

Since genetic testing suggested the presence of the RNF43 L122Tfs*3 mutation, we hypothesized that the RNF43 co-mutation might have prognostic implications. Previous research suggested RNF43 may be a cause of an inherited predisposition to CRC (12). Mutation-induced activation of Wnt-β-catenin signalling is a frequent driving event in tumours, especially in CRC, where APC mutation-induced activation account for approximately 67%. RNF43 gene is one of the negative feedback regulators in the WNT signalling pathway. Induced expression of RNF43 mediates ubiquitylation of WNT receptors, which drives their internalization and lysosomal degradation, thereby attenuating the sensitivity of cells to incoming WNTs, and the mutation rate in CRC is about 8% (13, 14). RNF43 and BRAF mutations are molecular events involved in serrated tumour pathways during CRC development (15). Previous studies have found that RNF43 mutations were mainly in the form of truncation, and associated with MSI-H and high value of TMB, and mutually exclusive with APC (14, 16, 17). To verify the effect of RNF43 mutation on the BRAF mutant CRC patients, samples from our cohort and public cohort were analysed. In terms of genomic characteristics, similar to the results of previous studies, the number of mutant genes, TMB value and proportion of MSI-H in the RNF43 mutant group were significantly higher than those in the wild-type group. The mutation rates of common and proven favourable genes were also significantly different between the two groups. We found that the mutation frequency of genes increased in RNF43 mutant group that have been shown to be associated with good prognosis, such as MLL, ARID1A, POLE and POLD1, while the TP53 and APC, two major drivers of CRC, were significantly decreased (2, 18–20). Lochlan et al. have found that APC gene mutation was an aggressive marker of BRAF mutant CRC, which may be associated with poor prognosis (21). In addition, because RNF43 indirectly regulates the WNT pathway, and RNF43 and APC mutations are mutually exclusive, we believe that RNF43 has a favourable effect on BRAF mutant CRC population compared with APC-driven oncogenic process. Although our data showed that there was no significant difference in the proportion of APC mutations between the two groups, we noticed that the APC mutations in the RNF43 mutation group were mainly missense mutations. Considering the proportion of truncation mutations, there was indeed a significant difference between the two groups. In addition, previous studies confirmed that RNF43 mutant CRC is enriched in CMS1 molecular subtype, a better prognostic subtype (13). Overall, based on previous articles and our results, we believe that in terms of genomic characteristics, CRC samples with BRAF and RNF43 co-mutation tend to have better prognostic genomic features, and RNF43 mutation may be possible to identify genomic features in BRAF mutant CRC samples.

Seeber et al. found that the DFS of the RNF43 mutation group was slightly lower than that of the wild-type group in CRC patients16. Matsumoto et al. found that RNF43 mutation is associated with aggressive tumour biology along with BRAF V600E mutation (22). But the prognostic impact of RNF43 mutation in BRAF mutant CRC patients remains unknown. In this study, DFS analysis was performed in 40 patients. Although there was a tendency to separate on the survival curve, no significantly difference was observed, probably because of the small number of outcome events. Meanwhile, in the analysis of public cohort, DFS still has no significant difference. However, further survival analysis showed that the OS and PFS of the RNF43 mutant group were significantly better than those of the wild-type group, and this effect was specific in the BRAF mutant patients. Considering that V600E is the most common BRAF mutation subtype (4), we separately analysed the prognostic impact of RNF43 mutation in the V600E mutant population. The difference in OS was more significant in the RNF43 mutant group, but PFS became insignificant. Recent biomarker analysis of the BEACON trial suggested the RNF43 mutation was associated with response to anti-BRAF/EGFR-based therapies but not in patients receiving standard chemotherapy± antiangiogenic agents (23). Ting Xu et al. also found that RNF43 mutation was enriched in BRAF V600E mutant mCRC patients who benefited from combination therapy with EGFR/BRAF inhibitors of different lines treatment (24). In the above PFS analysis of public cohort, we found that all patients with records were treated with chemotherapy± antiangiogenic agents as first-line treatment. Combined with the overall survival benefit of RNF43 mutant group, we suggested that RNF43 mutation possibly was a favourable marker for the prognosis of BRAF mutant mCRC population, regardless of the treatment regimen.

Similar to the function of RNF43, E3 ubiquitin ligase ZNRF3 is also an indirect negative feedback regulator of the WNT pathway. Nanhui Yu et al. have found that the high expression of ZNRF3 is associated with a good prognosis of CRC (25). However, whether its prognostic impact also remains unknown. In this study, we also analysed the impact of ZNRF3 mutation on the genomic characteristics and prognosis of BRAF mutant CRC patients from the public cohorts. The results were similar to those of RNF43. The number of mutations and MSI-H ratio in ZNRF3 mutant group were significantly higher than those in wild-type group, and PFS and OS were significantly longer than those in wild-type group. This also confirmed the effect of RNF43 mutation on BRAF mutant CRC patients.

The present study had several limitations. First, complete survival data in our prospective cohort is lacking, and the effect of RNF43 mutation on DFS is still unclear. Second, more samples are needed to confirm whether the prognostic effect of RNF43 mutation is consistent between BRAF V600E mutation and non-V600E mutation. Third, what kind of treatments can effectively respond to BRAF and RNF43 co-mutation patients also needs to be further confirmed. Recently et al. found that CRC patients with RNF43 mutation were sensitive to PIK3CA/mTOR inhibitors (26). At the same time, in terms of unique molecular landscape, RNF43 mutant tumours seems to be immune activated and may be sensitive to Wnt-targeted agents and immunotherapy16. Therefore, whether RNF43 mutation can provide further guidance for treatment (chemotherapy, targeted therapy or immunotherapy) in BRAF mutant mCRC needs to be explored. Nevertheless, we proposed that RNF43 mutation affects the genomic features of BRAF mutant CRC tumours and it can be used as a potential specific marker for future application.

## Data availability statement

The datasets presented in this study can be found in online repositories. The names of the repository/repositories and accession number(s) can be found below: The sequencing data has been successfully deposited in the Genome Variation Map. It can be found here: https://ngdc.cncb.ac.cn/gvm. The accession number is GVM000475.

## Ethics statement

Written informed consent was obtained from the individual(s) for the publication of any potentially identifiable images or data included in this article.

## Author contributions

LG contributed to the conceptualization and design. PS, JL, FS, and YZ contributed to the data curation and writing. WH, YH, and WS analysed and interpreted the data. All authors contributed to the article and approved the submitted version.
